# Redox Sensing within the Genus *Shewanella*

**DOI:** 10.3389/fmicb.2017.02568

**Published:** 2018-01-25

**Authors:** Howard W. Harris, Irene Sánchez-Andrea, Jeffrey S. McLean, Everett C. Salas, William Tran, Mohamed Y. El-Naggar, Kenneth H. Nealson

**Affiliations:** ^1^Department of Earth Sciences, Biological Sciences and Physics, University of Southern California, Los Angeles, CA, United States; ^2^Laboratory of Microbiology, Wageningen University, Wageningen, Netherlands; ^3^Department of Periodontics, University of Washington, Seattle, WA, United States; ^4^Microbial and Environmental Genomics, J. Craig Venter Institute, San Diego, CA, United States; ^5^Chevron, San Ramon, CA, United States

**Keywords:** redox sensing, MR-1, *Shewanella oneidensis*, energy taxis, extracellular electron transport, congregation, insoluble electron acceptors, dissimilatory

## Abstract

A novel bacterial behavior called congregation was recently described in *Shewanella oneidensis* MR-1 as the accumulation of cells around insoluble electron acceptors (IEA). It is the result of a series of “run-and-reversal” events enabled by modulation of swimming speed and direction. The model proposed that the swimming cells constantly sense their surroundings with specialized outer membrane cytochromes capable of extracellular electron transport (EET). Up to this point, neither the congregation nor attachment behavior have been studied in any other strains. In this study, the wild type of *S. oneidensis* MR-1 and several deletion mutants as well as eight other *Shewanella* strains (*Shewanella putrefaciens* CN32, *S*. sp. ANA-3, *S*. sp. W3-18-1, *Shewanella amazonensis* SB2B, *Shewanella loihica* PV-4, *Shewanella denitrificans* OS217, *Shewanella baltica* OS155, and *Shewanella frigidimarina* NCIMB400) were screened for the ability to congregate. To monitor congregation and attachment, specialized cell-tracking techniques, as well as a novel cell accumulation after photo-bleaching (CAAP) confocal microscopy technique were utilized in this study. We found a strong correlation between the ability of strain MR-1 to accumulate on mineral surface and the presence of key EET genes such as *mtrBC/omcA* (SO_1778, SO_1776, and SO_1779) and gene coding for methyl-accepting protein (MCPs) with *Ca*^+^ channel *che*motaxis receptor (Cache) domain (SO_2240). These EET and taxis genes were previously identified as essential for characteristic run and reversal swimming around IEA surfaces. CN32, ANA-3, and PV-4 congregated around both Fe(OH)_3_ and MnO_2_. Two other *Shewanella* spp. showed preferences for one oxide over the other: preferences that correlated with the metal content of the environments from which the strains were isolated: e.g., W3-18-1, which was isolated from an iron-rich habitat congregated and attached preferentially to Fe(OH)_3_, while SB2B, which was isolated from a MnO_2_-rich environment, preferred MnO_2_.

## Introduction

In the late 1980's, *Shewanella oneidensis* MR-1 (Myers and Nealson, 1988a) and later several species of *Geobacter* (Lovley et al., 1993; Champine et al., 2000) were shown to be capable of electron transfer to insoluble electron acceptors (IEAs), such as insoluble metal oxides and/or charged electrodes: a process called extracellular electron transport (EET) (Myers and Nealson, 1988b; Venkateswaran et al., 1999; Bond and Lovley, 2003). This ability attracted considerable interest with regard to biogeochemical cycling, bioremediation, corrosion, nano-materials processing, and energy production (Bretschger et al., 2007; Kan et al., 2011; Hsu et al., 2012). While several groups of microbes are known to be capable of EET, major mechanistic studies have been done with only two model systems, *Shewanella* (Fredrickson et al., 2008; Shi et al., 2009), and *Geobacter* (Lovley et al., 2004).

Thus far, more than 100 other strains of *Shewanella* have been isolated from a wide variety of habitats including open water column, sandstone shale, marine and fresh water sediments, oil-pipelines, oil brine, and even algal communities atop Antarctic Ice (Hau and Gralnick, 2007). The genomes of more than 20 of these species have been fully sequenced (Fredrickson et al., 2008). Several of these species have been shown to be capable of EET to IEA, including *S. oneidensis* MR-1*, Shewanella putrefaciens* CN32, *S*. sp. ANA-3, *S*. sp. W3-18-1, *Shewanella amazonensis* SB2B, *Shewanella frigidimarina* NCIMB 400, and *Shewanella loihica* PV-4 (Fredrickson et al., 1998; Venkateswaran et al., 1998; Gao et al., 2006; Bretschger, 2008). Other members of the *Shewanella* genus such as *Shewanella baltica* OS217 and *Shewanella denitrificans* OS155 (Table 1) are not capable of EET (Brettar et al., 2002). Of all these strains, the congregation in response to IEA has only been studied for MR-1.

**Table 1:** Genetic comparison of *Shewanella* spp. and their original habitat.

**Strain**	**MCP PAS like gene**	**MCP Cache like gene**	**mtrF like genes (SO_1780)**	**octaheme cytochrome-c like gene (SO_4142)**	**Habitat**	**References**
*S. oneidensis* MR-1	+	+	+	+	Sediment of lake Oneida, NY	Venkateswaran et al., 1999
*S. amazonensis* SB2B	+	+	+		Intertidal sediments of Amazon River delta, Brazil	Venkateswaran et al., 1998
*S. baltica* OS155		+			Oil brine water column of Baltic sea	Ziemke et al., 1998
*S. denitrificans* OS217					Oxic–anoxic interface of water column of Baltic Sea	Brettar et al., 2002
*S. frigidimarina* NCIMB 400		+			Water column of North Sea	Bowman et al., 1997
*S. putrefaciens* CN32	+	+		+	Shale sandstone in Albuquerque, New Mexico, USA	Fredrickson et al., 1998
S. sp. ANA-3	+	+	+	+	Arsenic-treated wooden poll in brackish water, Woods Hole, Massachusetts, USA	Saltikov et al., 2003
S. sp. W3-18-1	+	+		+	Iron-rich marine sediment, Washington coast, Pacific Ocean	Murray et al., 2001
*S. loihica* PV-4		+	+	+	Iron-rich microbial mat near a hydrothermal vent, Loihi Seamount, Pacific Ocean	Gao et al., 2006

**Figure 1:**
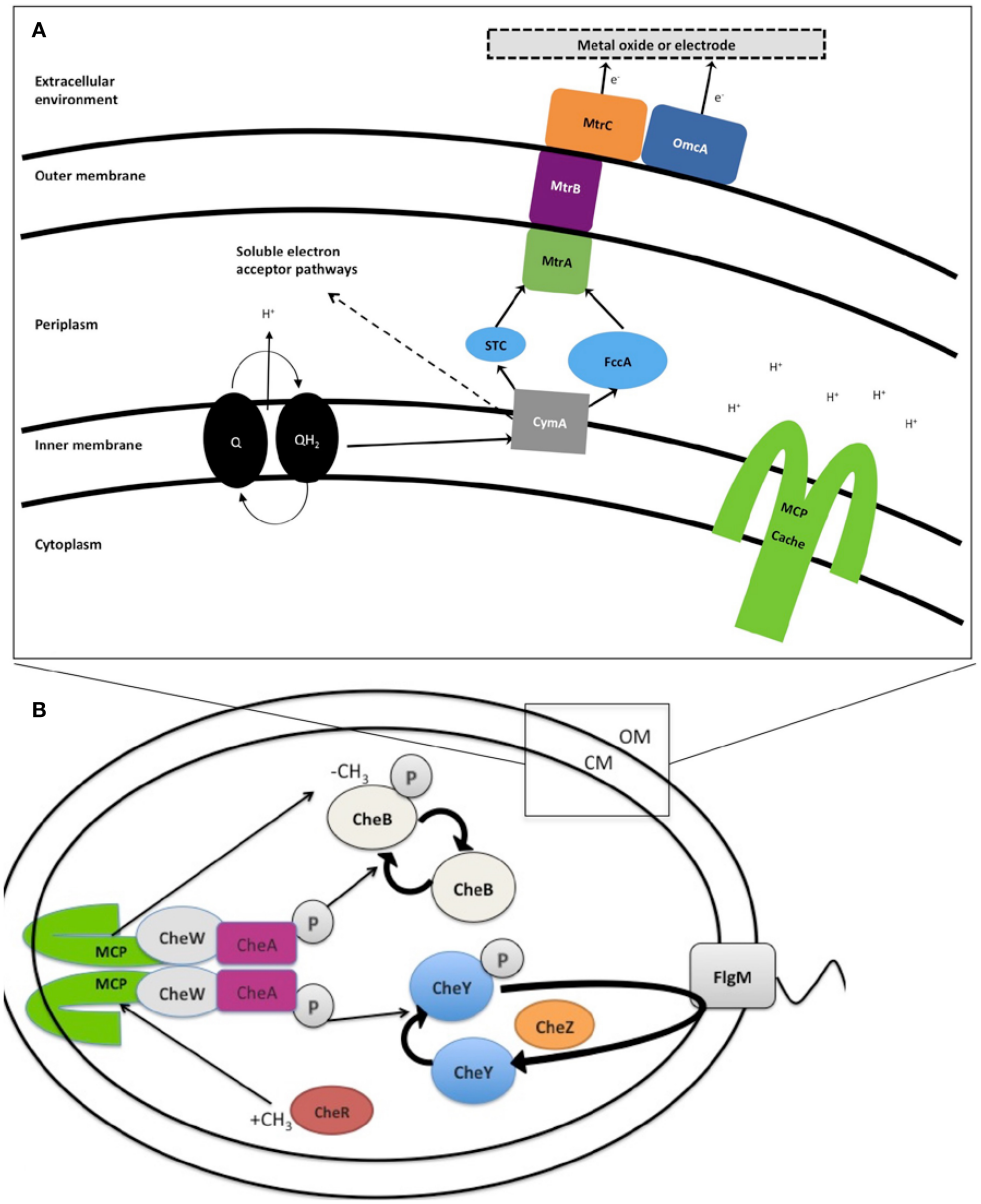
**(A)** Extracellular electron transport (EET) in *Shewanella oneidensis* MR-1 builds proton motive force (pmf). Under anaerobic conditions members of the genus *Shewanella* can transport electrons from the inner membrane, periplasm, outer membrane, and an electrode via a chain of cytochromes and menaquinones (MQ) known collectively as extracellular electron transport (EET). Expression of EET cytochromes can fluctuate based on the electron acceptor available in the environment. The number of iron containing *c*-type heme groups are indicated inside (parenthesis). Electron flows from electron donors, such as lactate, to reduce quinones (Q), which simultaneously transfer H^+^ into periplasm, building pmf, while also passing electrons to CymA. In the absence of soluble electron acceptors the electrons are transferred to MtrCAB outer membrane complex. This MtrCAB complex can donate electrons directly to terminal electron acceptor, either mineral or anode electrode, or via flavin molecules. The pmf drives the production of ATP and rotation of the polar flagella. “Self-sensing” methyl-accepting chemotaxis proteins (MCPs) control flagella rotation via the chemotaxis signal transduction system **(B)** and may detect changes in H^+^ concentration during metal reduction. **(B)** Likely chemotaxis signal transduction pathway in *Shewanella*. In response to stimulation, the structure of MCP shifts like a piston, causing the auto phosphorylation of CheA to slow or stop. CheY and CheB are, therefore, not phosphorylated, and this lack of CheY-P allows smooth swimming. This stimulation also has another effect—the CheB is inactive without phosphorylation (it cannot perform as a methylesterase) and this allows for the CheR protein (a continually active methyltransferase) to outcompete and freely methylate the dimer methyl-accepting region of MCP (HAMP domain). This methylation of the MCP acts to increase the auto-phosphorylation rate of CheA Histidine residue. Therefore, the signal transduction system has control over the flagellar reversal frequency in the presence of increased or decreased stimuli, leading to a series of “run-and-reversal” swimming.

**Figure 2:**
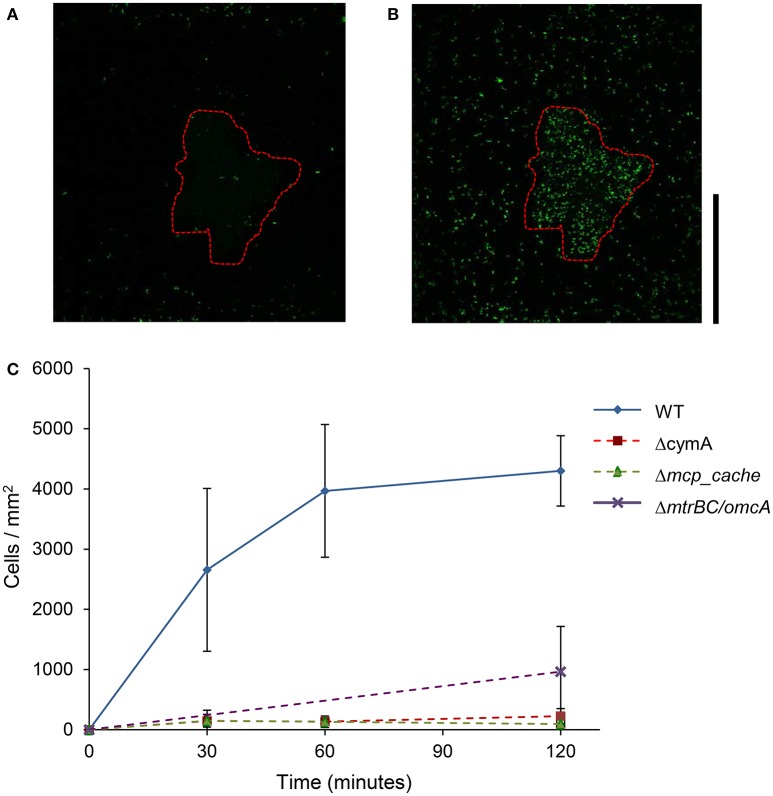
Swimming MR-1 can migrate toward insoluble electron acceptor minerals and attach. Representative confocal fluorescence microscopy image of WT MR-1 cells at *t* = 0 and *t* = 120 min **(A,B)**. Swimming WT MR-1 GFP cells were introduced to MnO_2_ particle (red dotted outline) in anaerobic sealed capillary. At *t* = 0 all the cells were irreversibly *photo-bleached* in a 250 by 250 μm area around particle **(A)**. Fluorescent cells from outside bleached zone that swim into frame and attach to mineral surface were then counted. The black vertical scale bar on the right represents 100 μm. Graph **(C)** compares WT MR-1 attachment on MnO_2_ particle over 120 min with chemotaxis and extracellular electron transfer deletion mutants (MR-1 Δ*mcp_cache*, Δ*cymA*, and Δ*mtrBC/omcA*). The error bars include 2 std deviations.

Within the genus *Shewanella*, the EET mechanism of MR-1 has been the most extensively characterized. MR-1 employs several approaches for insoluble IEAs reduction: (1) direct EET via extracellular multiheme cytochromes (Beliaev and Saffarini, 1998; Myers and Myers, 2001, 2002; Meyer et al., 2004; Mitchell et al., 2012; Kracke et al., 2015) (Figure 1A); (2) mediated EET using soluble electron shuttles bound to membrane cytochromes (Lovley et al., 1996; Marsili et al., 2008; Li et al., 2012; Kotloski and Gralnick, 2013; Okamoto et al., 2014); (3) mediated EET utilizing conductive outer membrane extensions that contain cytochromes (Gorby et al., 2006; El-Naggar et al., 2010); and (4) conductive extracellular matrices containing conductive and semiconductive minerals (Kato et al., 2010). Several genes have been identified in strain MR-1 and shown to be essential for EET (Figure 1A and Table 2), including the tetraheme cytochrome *c cymA* (SO_4591) and the combination of *mtrBC/omcA* (SO_1776, SO_1778, and SO_1779) that code for the decaheme cytochrome *c* component and tetraheme cytochrome *c* necessary for reduction of several anaerobic electron acceptors, including metal oxides (Myers and Myers, 2001, 2002; Schwalb et al., 2003). Because all these mechanisms rely on the cell proximity to IEA for EET, it is important to understand the cell sensing and net swimming migration toward the IEA. With regard to congregation, redox taxis or energy taxis, many studies have been conducted on MR-1 due to its versatile electron acceptor utilization (Bencharit and Ward, 2005; Baraquet et al., 2009; Harris et al., 2010). Energy taxis is a term that broadly encompasses aerotaxis, phototaxis, redox taxis, taxis to alternative electron acceptors, and chemotaxis to oxidizable substrates (Alexandre et al., 2004).

**Table 2:** Genes of MR-1 described in the text.

**Gene name**	**Locus tag**	**Description**	**Role**	**References**
*cymA*	SO_4591	Tetraheme cytochrome *c*	Necessary for reduction of several anaerobic electron acceptors, including metal oxides	Myers and Myers, 1997; Schwalb et al., 2003
*NA*	SO_4142	Periplasmic monoheme cytochrome c	Unknown	
*mtrC*	SO_1778	Surface decaheme cytochrome c component	Extracellular metal oxide respiration	Coursolle and Gralnick, 2010
*mtrF*	SO_1780	Decaheme cytochrome c component	Unknown	
*mtrB*	SO_1776	Periplasmic EET component	Extracellular metal oxide respiration	Beliaev and Saffarini, 1998
*omcA*	SO_1779	Surface decaheme cytochrome c component	Extracellular metal oxide respiration	Beliaev and Saffarini, 1998; Myers and Myers, 2001
*mtrBC/omcA*	SO_1778, SO_1776, SO_1779	Outer-membrane decaheme *c*-type cytochromes and periplasmic EET component	Extracellular metal oxide respiration	Myers and Myers, 2001; Coursolle and Gralnick, 2010
*cheA-3*	SO_3207	Histidine protein kinase	Chemotactic signal transduction	Li et al., 2007; Coursolle and Gralnick, 2010
*mcp cache*	SO_2240	MCP with a Cache domain	Energy taxis in response to soluble electron acceptors and congregation	Baraquet et al., 2009
*mcp pas*	SO_1385	MCP with PAS domain	Energy taxis and congregation around Fe(OH)_3_	Baraquet et al., 2009; Harris et al., 2012

It is well-documented that the accumulation of MR-1 cells in response to soluble electron acceptors is a form of energy taxis, which depends on H^+^ flux and the establishment of a proton motive force (Baraquet et al., 2009) (Figure 1). A part of this response includes more rapid swimming, as also seen with electron shuttles such as riboflavin or anthraquinone 2.6-disulfonate (AQDS) (Bencharit and Ward, 2005; Harris et al., 2010; Li et al., 2012). In contrast, the accumulation of cells around IEA, which has been called congregation (Nealson et al., 1995), involves both increased swimming speed upon contact with the IEA (called electrokinesis), and increased swimming reversals upon a decrease in PMF. Swimming reversals allow multiple transient cell-IEA encounters (lasting for 1–100 ms), and the rate at which swimming cells transition to irreversible attachment to IEA during congregation has not yet been quantified.

The mechanism(s) that cells utilize to locate IEAs remain unclear (Nealson et al., 1995; Bencharit and Ward, 2005; Harris et al., 2010). Early reports proposed that the other studied model organism *Geobacter*, accumulates around IEAs by sensing a gradient of reduced metal ions (Childers et al., 2002), however, reduced metal ions are not involved with the sensing mechanism used by MR-1 (Bencharit and Ward, 2005). Bacterial congregation in response to poised electrodes was recently described, pointing to the redox sensing, rather than metal ion sensing, as the trigger for response (Harris et al., 2010). The positive applied potentials to electrode (200–600 mV vs. Ag/AgCl) caused MR-1 to congregate similar to that seen with metal oxides (Harris et al., 2012). Redox potentials of MnO_2_ containing minerals range between 400 and 600 mV vs. Ag/AgCl and accept electrons more readily than Fe(OH)_3_ minerals, which carry the equivalent poised potential of 100 to 300 mV vs. Ag/AgCl (Burdige, 1993). In a previous study, the characteristic swimming of MR-1 around IEA was hypothesized to be regulated by two self-sensing chemotaxis receptors, methyl-accepting proteins (MCPs) with, *Ca*^+^ channel *che*motaxis receptor (Cache) domain (SO_2240) and Per/Arnt/Sim (PAS) domains (SO_1385), and by the chemotaxis signal transduction protein kinase CheA-3 (SO_3207) to allow the cell to sample the redox potential, or electron accepting ability of a surface (Table 2 and Figure 1B) (Harris et al., 2012). Before this study, the genes responsible for the motility driven attachment of cells on and around IEA were unknown.

In this report, we study the congregation mechanism of different *Shewanella* strains by monitoring swimming patterns and cell attachments to MnO_2_ and Fe(OH)_3_. In addition to screening the WT and several deletion mutants of MR-1, other *Shewanella* species (see Table 1) were screened. Many strains were shown to be capable of congregation around both MnO_2_ and FeOH_3_, while others responded selectively to MnO_2_ (SB2B) or Fe(OH)_3_ (W3-18-1). When cell attachment to the mineral surfaces was monitored we observed a strong correlation between the ability of the cell to congregate, and the attachment of the cells to the IEA surface. We then compared the genomes of these species to find candidate genes involved in the congregation swimming, accumulation, and cell attachment phenotypes in response to specific IEA surface.

## Results

### Cell accumulation and attachment to mineral requires chemotaxis and extracellular electron transport genes in *S. oneidensis* MR-1

Most of the early studies of energy taxis in MR-1 utilized a method of swarm plate assays (Nealson et al., 1995; Baraquet et al., 2009; Li et al., 2012). Here, we offer a more in-depth characterization of these yet unknown energy taxis mechanisms by using a quantitative method of tracking the swimming of individual cells through liquid media in response to IEA and cell accumulation after photo-bleaching (CAAP) to measure cell attachment rate (Li et al., 2010). CAAP utilizes irradiation from a confocal microscope UV laser to irreversibly darken (quench the fluorescence of) GFP-labeled cells on and around a given IEA. By quantifying fluorescent cells as they move into the darkened zone, the rates of accumulation and attachment can be quantified, and different strains compared. To be considered attached, the position of the motile cell is verified by analyzing a 3D image of the field of view. If the cell comes to rest on the mineral surface then the cell is then considered “attached.” The strength of bonds between stationary MR-1 cells and surfaces was measured with optical tweezers in a separate study (Gross and El-Naggar, 2015). This method allows distinction between directed cell-attachment, and random electrostatic attachment.

As shown in Figure 2, the WT MR-1 cells attached to mineral surface after 30 min with some large deviation in number, 2,655 ± 1,352 cells/mm^2^. During this time, the cells exhibit “touch and go” swimming, making transient contact with the MnO_2_ mineral surface. After 2 h, the number of new cells attached to the mineral surface increased to 4,300 ± 584 cells/mm^2^ of mineral surface while additional motile cells continued to congregate (Videos S1, S2, and S17). Mutants with triple deletions in key EET genes *mtrBC/omcA* (SO_1776, SO_1778, SO_1779), single deletion of *cymA* (SO_4591) and the major energy taxis chemoreceptor MCP Cache (SO_2240) have been previously linked to swimming congregation phenotype (Harris et al., 2012). Deletion mutants (Δ*cymA*, Δ*mcp_cache*, and Δ*mtrBC/omcA*) were all motile and capable of reversing swimming direction, but were incapable of congregation and showed little or no attachment to MnO_2_ during the experiment (Figure 2C and Figures S1A,B). WT accumulation in bleached zone at *t* = 2 h averages 4,300 ± 584 cells/mm^2^ while Δ*mcp_cache*, and Δ*mtrBC/omcA* mutants accumulate in negligible numbers (Figure 2C and S1CD).

### Characterizing congregation around IEA in other *Shewanella* strains

Nine strains of *Shewanella* were tested for their ability to congregate around mineral surfaces of MnO_2_ and Fe(OH)_3_ with time series assay of cell attachment to mineral (Table 3 and Videos S3–S14). As with MR-1, all nine strains examined had a single polar flagellum and reversal of swimming direction was accomplished by reversal of flagellar rotation (data not shown). The swimming tracks within the same experiment were sorted into two separate groups based on swimming path (Table 3): those that contacted insoluble metal oxide surface (swam within 2 μm) compared with those that did not contact (swam >2 μm). Contacting swimmers that demonstrated significant increase in reversal frequency and swimming velocity than non-contacting group (*P* < 0.05) are classified as positive for congregation behavior as designated with superscript letters (Table 3).

**Table 3:** Bacteria swimming speed and reversal frequency around metal oxide minerals.

**Strain**	**Mineral**	**Reversal frequency (reversals/s)**		**Speed (μm/s)**	
		**≤2 μm**	**>2 μm**	**≤2 μm**	**>2 μm**
MR-1	MnO_2_	0.97 ± 0.58^a^	0.32 ± 0.48^a^	24.37 ± 6^k^	19.26 ± 11.2^k^
	Fe(OH)_3_	0.74 ± 0.5^b^	0.21 ± 0.39^b^	18.12 ± 5.4^l^	12.6 ± 5.4^l^
SB2B	MnO_2_	1.657 ± 0.925^c^	0.320 ± 0.462^c^	37.7 ± 14.7^m^	23.5 ± 8.79^m^
	Fe(OH)_3_	nr	nr	nr	nr
PV-4	MnO_2_	0.930 ± 0.3^d^	0.519 ± 0.7^d^	56.05 ± 35.8^n^	48.49 ± 59.8^n^
	Fe(OH)_3_	0.177 ± 0.34^e^	0.586 ± 0.59^e^	12.73 ± 6.1	13.57 ± 4.4
W3-18-1	MnO_2_	nr	nr	nr	nr
	Fe(OH)_3_	0.228 ± 0.39	0.298 ± 0.27	15.54 ± 9.7^p^	9.48 ± 1.5^p^
CN32	MnO_2_	1.371 ± 0.98^g^	0.622 ± 0.49^g^	34.98 ± 10.18^q^	22.6 ± 8.4^q^
	Fe(OH)_3_	0.573 ± 0.47^h^	0.342 ± 0.39^h^	17.86 ± 6.5^r^	13.62 ± 5.1^r^
ANA3	MnO_2_	1.240 ± 0.91^i^	0.416 ± 0.47^i^	20.38 ± 3.7^s^	14.71 ± 6.7^s^
	Fe(OH)_3_	0.786 ± 0.45^j^	0.426 ± 0.49^j^	21.79 ± 7.7^t^	13.87 ± 5.7^t^

Results

a−t indicates significant difference of ± 2 S.D.

*nr = no response. OS155, NCIMB400, and OS217 did not show response to minerals*.

**Figure 3:**
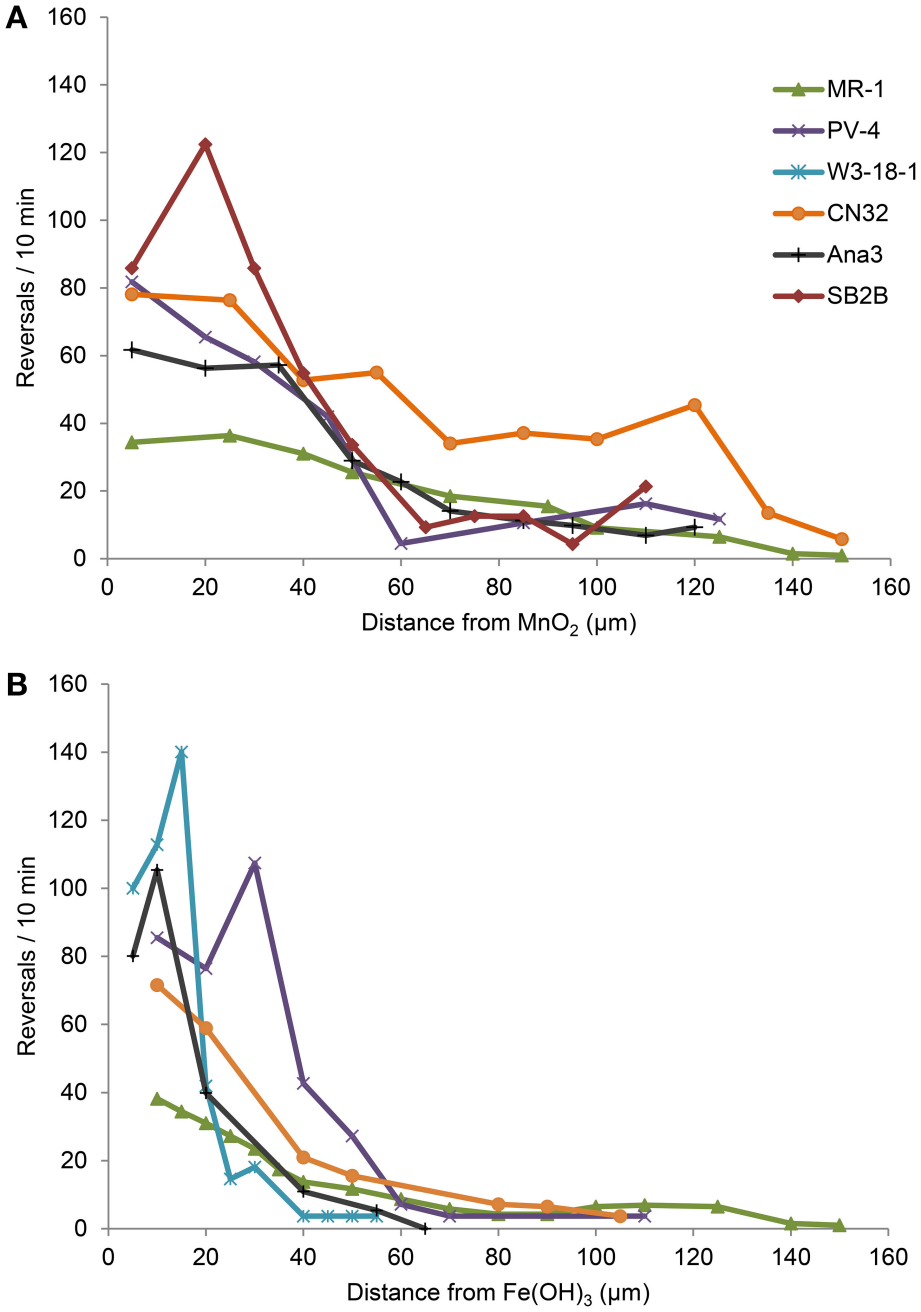
**(A,B)** Reversal frequency (y-axis) of swimming *Shewanella* cells vs. distance (x-axis) from IEA particle. The average reversal frequencies of individual tracked swimming cells are divided into bins of 5 μm along the x-axis for experiments with MnO_2_ **(A)** or Fe(OH)_3_ particle **(B)**.

Strains ANA3, CN32, and PV-4, in addition to MR-1, were positive for congregation around both MnO_2_ and Fe(OH)_3_ (Table 3, Figure 3; Videos S1–S8). They showed maximum reversal rates when they were located between 5 and 40 μm from the MnO_2_ or FeOH_3_ particle surface (Figure 3). Three strains: OS217, NCIMB400, and OS155 were not motile at time *t* = 30 min—in response to Fe(OH)_3_ or MnO_2_ minerals (Table 3). For example, the reversal frequency of the swimming MR-1 cells contacting MnO_2_ was 0.94 ± 0.53 reversals/s, while the reversal frequency of the non-contacting cells was 0.62 ± 0.73 reversals/s. The speed also increased in the contacting group of MR-1 from 19.26 ± 11.2 μm/s in the non-contacting group to 24.37 ± 6 μm/s in the contacting group. Because there was both a statistically significant increase in swimming speed and reversals so as to allow the cells to remain near the metal oxide particle this strain was said to be congregation positive.

**Figure 4:**
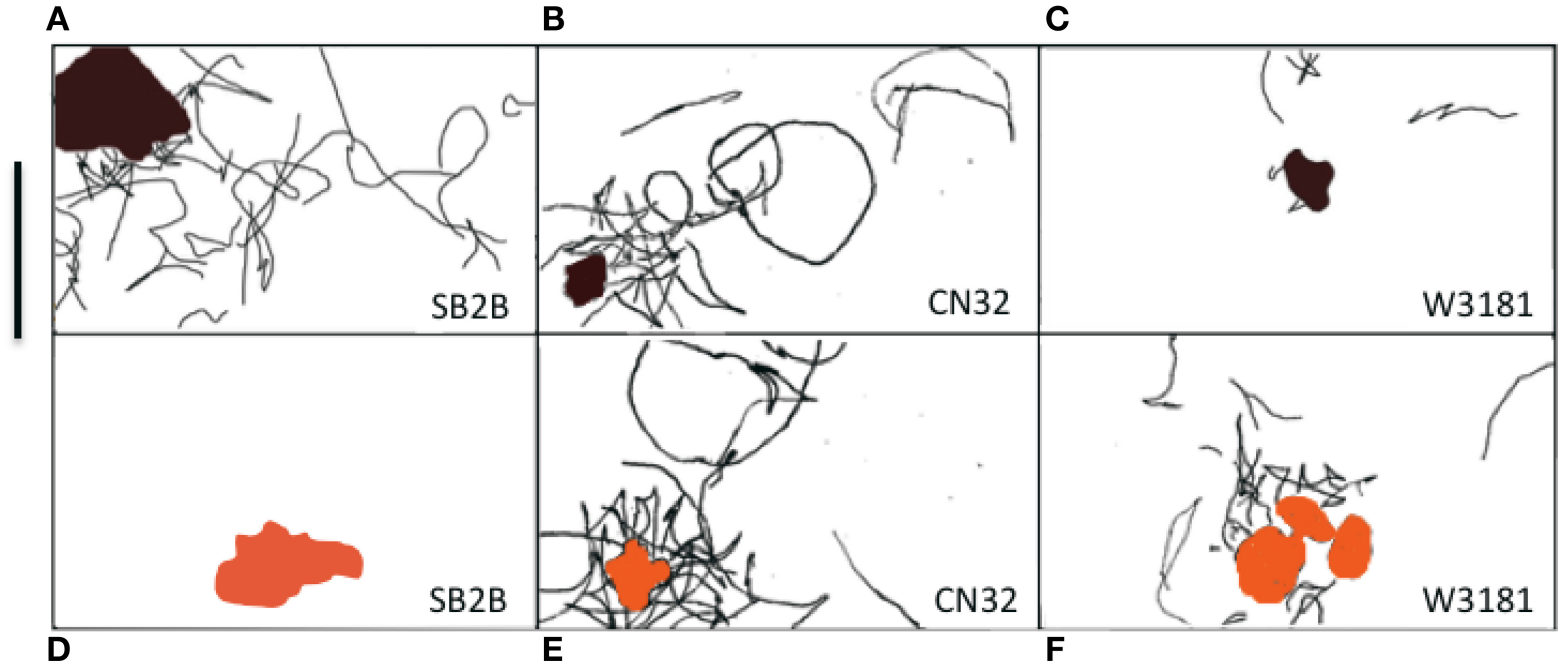
**(A–F)** Panel of three *Shewanella* spp. swimming tracks (30 s) demonstrated a preference for metal oxide minerals. The swimming behavior of three motile *Shewanella* spp. in response to MnO_2_ (top row—black colored particle) and Fe(OH)_3_ (bottom row—orange colored particle) was studied. Congregation occurs in anaerobic conditions, swimming tracks are shown in black. SB2B (left), CN32 (center), and W3181 (right). The cell swimming was tracked by hand. The scale bar on the upper left = 50 μm. The brown color shape represents MnO_2_ and the orange represents Fe(OH)_3_. When there are no motile cells detected this is indicated by the absence of black lines.

*Shewanella* spp. swimming tracks (30 s) that demonstrated a preference for metal oxide minerals are highlighted in Figures 4A–F. Figure SB2B cells displayed no swimming response to Fe(OH)_3_ (Figure 4D) while exhibiting active congregation around MnO_2_ particles (Figure 4A). In contrast, W3-18-1 cells congregated around Fe(OH)_3_ particles (Figure 4C) but showed significantly diminished swimming and reversal activity around MnO_2_ (Figure 4F; Videos S9, S14).

## Discussion

Members of the genus *Shewanella* are comprised of heterotrophic, facultative aerobes capable of utilizing a wide range of organics and inorganics as energy sources. Many *Shewanella* strains have been isolated from water column and sediment habitats in locations all across the globe. In addition to soluble electron acceptors, many of these organisms can respire a wide assortment of naturally occurring insoluble metal oxides under anoxic conditions.

In our study of congregation behavior in eight *Shewanella* species, we used cell tracking with computer analysis and time series assay of cell attachment to MnO_2_ or Fe(OH)_3_ to demonstrate that five out of eight shewanellae were capable of this behavior CN32, ANA-3, W3-18-1, SB2B, and PV-4) and that some strains (W3-18-1, SB2B) show a preference for one metal oxide over the other under these conditions (Figure 4). Such findings are consistent with the notion that these bacteria have adapted to the prevalent insoluble electron acceptor found in the habitat from which they were isolated. Furthermore, three *Shewanella* strains that were isolated from the water column did not congregate in response to IEA. Whether these differences derive from the absence of genes involved in EET (as in the OS217) or other reasons will be a point for future studies.

In other studies, overnight growth of the *Shewanella* strains comparing reduction rates of various metal oxides in head to head comparison, have been shown to reduce metal oxide preferentially from their environmental niche (Bretschger et al., 2007). The results presented here are consistent with the hypothesis that *Shewanella* species have evolved a congregation and attachment behavior consistent with the environments from which they were isolated (Table 3). For example, W3-18-1 seems to reveal significant inclination for congregation in the presence of and attachment to Fe(OH)_3_ minerals native to Pacific continental shelf (Harris et al., 2012) while SB2B prefers MnO_2_ (Venkateswaran et al., 1998) similar to that of the Amazon river sediment.

The genomic comparison in Table 1 suggests that the presence of octaheme cytochrome (SO_4142) may be important for swimming in response to relatively “low” redox potentials (100–300 mV vs. Ag/AgCl) of Fe(OH)_3_. While *mtrF* (SO_1780) may be needed for response to relatively “higher” redox potentials of MnO_2_ (between 400 and 600 mV vs. Ag/AgCl). This hypothesis could then be tested with deletion mutants in MR-1. This work complements previous work of Harris et al. (2012), by revealing that genes involved with the net effect of the motility behavior toward IEA minerals or electrodes is cumulative through relevant time scales of 1–30 min (Figure 2) (Harris et al., 2012). Our findings with CAAP confocal microscopy technique, demonstrated that congregation leads to a slow migration of cells toward IEA minerals with eventual attachment. Thus, this behavior is appropriately termed congregation, as it describes the gathering or accumulation of motile bacteria around IEA. We showed here that genes *mcp cache* and *mtrBC/omcA* are essential for accumulation and attachment phenotype in MR-1, in addition to being responsible for the characteristic swimming patterns of increased speed and run-and-reversal type behavior that was identified previously (Harris et al., 2012).

The results of the experiments with Δ*cymA*, Δ*mtrBC/omcA*, and Δ*mcp_cache* mutants in MR-1 show an inhibition of accumulation, attachment and congregation behavior in response to all IEAs. Therefore, it can be hypothesized that the presence of homologous EET genes (*cymA, mtrB, mtrC, omcA*), and methyl accepting chemotaxis gene (*mcp_cache*) determines the phenotypic responses we see in other *Shewanella* spp. (Table 3). Genetic comparison of strains, which could respond to “lower” redox potential IEA, indicates that peripheral outer membrane octaheme cytochromes (such as SO_4142) may play some role in responding to “lower” redox potential IEA. Hence these observations on sustained swimming around specific minerals corresponded with specific genotypes of the different species.

During many MFC (Kotloski and Gralnick, 2013) and metal reducing batch culture experiments, extracellular electron transfer-mediated energy taxis, or congregation ability, was not measured (Kotloski and Gralnick, 2013). In metal oxide reduction assays the 3D distribution of cells and cell motility could greatly influence metal reduction due to incubator shaker speed, culture flask dimensions, or mixing. Congregation behavior directly influences cell attachment to IEAs. The relationship between congregation and nanowire-like appendage formation is still unknown (Pirbadian et al., 2015), although the motility and congregation parameters are rarely monitored in these studies (Gorby et al., 2005; Reguera et al., 2005). Transforming these core congregation genes (*cymA, mtrB, mtrA, omcA, mcp_pas*, and *mcp_cache*) into other bacteria species, with single polar flagellum, may someday improve bioremediation capabilities by being able to induce bacterial attachment and colonization of surfaces that would otherwise be difficult or impossible.

## Materials and methods

### Cultivation and strains

MR-1 and several deletion mutants originated from MR-1 were examined in this study (Table 2) (Beliaev and Saffarini, 1998; Myers and Myers, 2002). Strains were inoculated from glycerol stocks stored at −80°C onto Luria-Bertani (LB) plates and grown overnight at 30°C. Individual colonies were then selected and inoculated into 5 mL of defined minimal media (M1) (Bretschger et al., 2007) supplemented with 18 mM lactate as an energy source (Bretschger et al., 2007) in 15 mL tubes (VWR International LLC, Randor, Pennsylvania, USA) and incubated horizontally in a shaker (180 rpm) for 48 h at 30 °C. Optical Density was measured using a spectrophotometer (Unico 1100RS spectrophotometer, Dayton, New Jersey, USA). Cells were sampled at an OD_600_ of 0.5 ± 0.2 (after ~48 h). In swimming experiments, five milliliter cultures were sampled when the cells reached an OD of 0.4, mixed with manganese or iron oxides, and introduced to a glass capillary (0.02 × 0.20 mm) (Vitrocom, Mountain Lakes, New Jersey, USA) that was then sealed using vacuum grease as described previously (Harris et al., 2010).

### Mineral synthesis

The Fe(OH)_3_ stock solution was prepared according to the protocol by Cornell and Schwertmann and then verified by X-ray defraction (Schwertmann and Cornell, 2008). This preparation of colloidal MnO_2_ began with 8 g KMnO_4_ dissolved in 200 mL, while utilizing all possible safety precautions. The solution was continuously mixed using a magnetic stir bar on high and heated below boiling temperature. Then, 5 mL of 10 M sodium hydroxide was added to neutralize the acid produced by the reaction. In a separate flask, 15 g of manganese chloride was dissolved into 75 mL of distilled water. Finally, the solution was then slowly mixed with the permanganate solution (in a chemical fume hood) for 75 min. After cooling the solution, it was then washed by centrifugation and rinsed with deionized water (DI) (18 Meg-Ohm cm) water over five times. The final precipitate was allowed to dry by vacuum filter in a clean bench and desiccated for 36 h. The resulting minerals were analyzed via X-ray diffraction to confirm the production of Fe(OH)_3_ and MnO_2_ (Bretschger et al., 2007; Salas et al., 2010).

Suspended mineral particles were mixed with culture at a final concentration of 300 mg/mL of MnO_2_ or Fe(OH)_3_. Cells were then immersed by capillary into rectangular capillary tubes (0.02 × 0.20 mm) (Vitrocom, Mountain Lakes, New Jersey, USA). Tubes were sealed with Silicon vacuum grease (Dow Corning, Midland, Milwaukie, USA) and observed with light microscopy, fluorescence microscopy, and confocal microscopy.

### Cell accumulation after photo-bleaching (CAAP) time-lapse experiments

All fluorescently labeled strains (GFP) were transformed as previously described (McLean et al., 2008) and then grown aerobically on a defined minimal medium with 25 μg/ml kanamycin and 18 mM lactate for 48 h at 30°C. Five milliliter cultures were sampled when the cells reached an OD of 0.4, mixed with manganese or iron oxides, and introduced to a glass capillary (0.02 × 0.20 mm) (Vitrocom, Mountain Lakes, New Jersey, USA) that was then sealed using vacuum grease as described previously (Harris et al., 2010). GFP labeled WT MR-1, Δ*cymA*, Δ*mcp_cache*, and Δ*mtrBC/omcA* cells were bleached using maximum light intensity settings with 60X lenses, of an inverted Leica TCS SPE confocal microscope (Wetzlar, Germany) for 15 min. To ensure that bleaching occurred, time-lapse captured a screen area (250 × 250 μm selected area) every minute until the original cells appeared dark and the surrounding cells remained brightly fluorescent. Images were then captured using 588 nm excitation and 530 nm emission. A time-lapse video of the entire section of the tube was captured using Leica Imaging software and the “auto focus” feature for the next 3 h. Cells were also observed under transmitted light mode to verify that bleached cells were motile and intact. A separate negative control, with GFP labeled Δ*mcp_cache*, was captured for 3 h. No cells were seen accumulating in the dark zone in this negative control, nor did bleached cells recover GFP fluorescence. The response of the entire capillary (height of 30 μm) was captured using time-lapse photography and the sum of cells in all 20 z-axis stacks was determined (using computer analysis method below) for each time point.

### Microscopy capture of cell movements

The methods for bacterial tracking and analysis were identical to previous studies utilized for Figure 3 and recorded in Table 3 (Harris et al., 2012). Both computer and manual bacterial swimming tracks were standardized using a scale ruler (100 μm). From each experiment, the overall swimming activity within the video frame—equivalent to a 107 × 193 μm field of view—was recorded and the video was time-normalized to give swimming speeds in μm/s. Several parameters were measured for each bacterial swimming track such as the total distance moved, the time of track since the bacteria first appear and disappear, the number of reversals, the distance between each reversal and the metal oxide, and the distance between the metal oxide and the start of the bacteria track.

### Swimming analysis

Cells were tracked by hand from video data (30 frames/s), frame-by-frame, and measured by ImageJ image analysis software. Data inputs include the swimming speed, the starting position of the bacteria with respect to the nearest IEA surface and position of each bacterial reversal event was identified and logged with respect to the distance from the nearest IEA surface (Figure 3 and Table 3). For determining the swimming activity after contact with IEA, the swimming cells were divided into two groups for analysis: cells that swam within 2 μm of a particle were considered “contacting” and those that did not swim within 2 μm from the particle surface were considered “non-contacting.” In addition to the hand tracking methods described above, experimental data was then computer sorted and analyzed by an algorithm previously described to yield the calculated swimming data (Crocker and Grier, 1996; Harris et al., 2012; Harris, 2013). To produce Figures 3, 4, all experimental data from four separate biological replicates were combined, pooled and averaged into bins according to distance from the IEA particle. Because this data was pooled into distances, error bars are overlapping; no comparison of reversal frequency, at a given distance can be made between strains, as this method of visualization precludes experiment identifiers.

## Author contributions

HH: designed and performed the experiments; HH and IS-A: wrote and revised paper; ES, ME-N, and JM: helped design experiments; WT: helped to perform the data analysis; JM and KN: revised the manuscript.

### Conflict of interest statement

The authors declare that the research was conducted in the absence of any commercial or financial relationships that could be construed as a potential conflict of interest.
