# Supplementary material for: Redox Sensing within the Genus Shewanella
Source: Front Microbiol. 2018 Jan 25;8:2568. doi: 10.3389/fmicb.2017.02568 (PMC5789149; doi:10.3389/fmicb.2017.02568)
Supplement: Supplementary file 1 [file Presentation1.pdf]

SUPPLEMENTAL MATERIALS

FIGURE S1. CAAP experiment with Δ*mtrAB*/*omcA* and Δ*SO_2240* fluorescent motile cells near MnO_2 after photo-bleaching.

Left column shows GFP labeled cells at t = 0 while right column shows experiment at time = 2 h. Significant accumulation and attachment occurs in WT MR-1. EET deletion mutants Δ*mtrAB*/*omcA* (B) and methyl accepting chemotaxis mutant Δ*SO_2240* (C) do not accumulate or attach in significant numbers relative to WT. The black vertical scale bar on bottom right represents 100 µm.

All Supplemental Video files can be downloaded here: https://figshare.com/s/700c93156db04ddbdd7f

VIDEO S1**. Top view time-lapse video of wild type MR-1 cells during CAAP experiment.** MR-1 cells false colored to represent z-axis contour with red top 30µm; blue 0 µm bottom, during accumulation and attachment on MnO_2 (purple), over 1 hour and 30 min. In final still frame, MR-1 cells are colored green.

VIDEO S2**. Revolving 3D axis perspective of time-lapse video of wild type MR-1 cells.** MR-1 cells are false colored to represent z-axis contour with red top 30µm; blue 0 µm bottom, during accumulation and attachment on MnO_2 over 1 hour and 30 min.

VIDEO S3**. MR-1 congregation behavior around MnO_2.**

VIDEO S4**. MR-1 congregation behavior around $Fe(OH)_3$.**

VIDEO S5**. SB2B congregation behavior around MnO_2.**

VIDEO S6. **SB2B behavior around $Fe(OH)_3$.**

VIDEO S7. **CN32 congregation behavior around MnO_2.**

VIDEO S8. **CN32 congregation behavior around $Fe(OH)_3$.**

VIDEO S9. **W318-1 behavior around MnO_2.**

VIDEO S10. **W318-1 congregation behavior around $Fe(OH)_3$.**

VIDEO S11. **Ana3 behavior around MnO_2.**

VIDEO S12. **Ana3 behavior around $Fe(OH)_3$.**

VIDEO S13. **PV4 behavior around MnO_2.**

VIDEO S14. **PV4 behavior around $Fe(OH)_3$.**

VIDEO S15. **Time-lapse bacterial reduction of MnO_2.** MR-1 cells can be seen swimming at speeds of 40–80 μm/s around an MnO_2 particle as it is being reduced. The bacteria for this experiment were sealed in an anaerobic capillary tube, with LB, ~15 min before recording. The tube remained sealed until the MnO_2 particle was fully reduced. Video was recorded using a combination of ×40 and ×100 optical microscopy to allow observation of the particle reduction and the microbial response respectively. Originally published in (2010) Proc. Natl. Acad. Sci. U.S.A.

VIDEO S16. **Time-lapse bacterial reduction of $Fe(OH)_3$.** MR-1 cells can be seen swimming at speeds of 40–80 μm/s around an $Fe(OH)_3$ particle as it is being reduced. The bacteria for this experiment were sealed in an anaerobic capillary tube, with LB, ~15

min before recording. The tube remained sealed until the $Fe(OH)_3$ particle was partially reduced. Video was recorded using a combination of ×40 and ×100 optical microscopy to allow observation of the particle reduction and the microbial response respectively. Originally published in Harris, (2010) Proc. Natl. Acad. Sci. U.S.A.

VIDEO S17**. Top view time-lapse video of wild type MR-1 cells during CAAP experiment.** Video, representing 3 hours of real time, was captured using phase contrast and fluorescent confocal microscopy (600x total magnification). MR-1 cells are labeled with GFP, were sealed inside anaerobic capillary tube containing MnO_2, minimal media and 18mM lactate. Originally published in Harris, (2012) Biochemical Transactions.

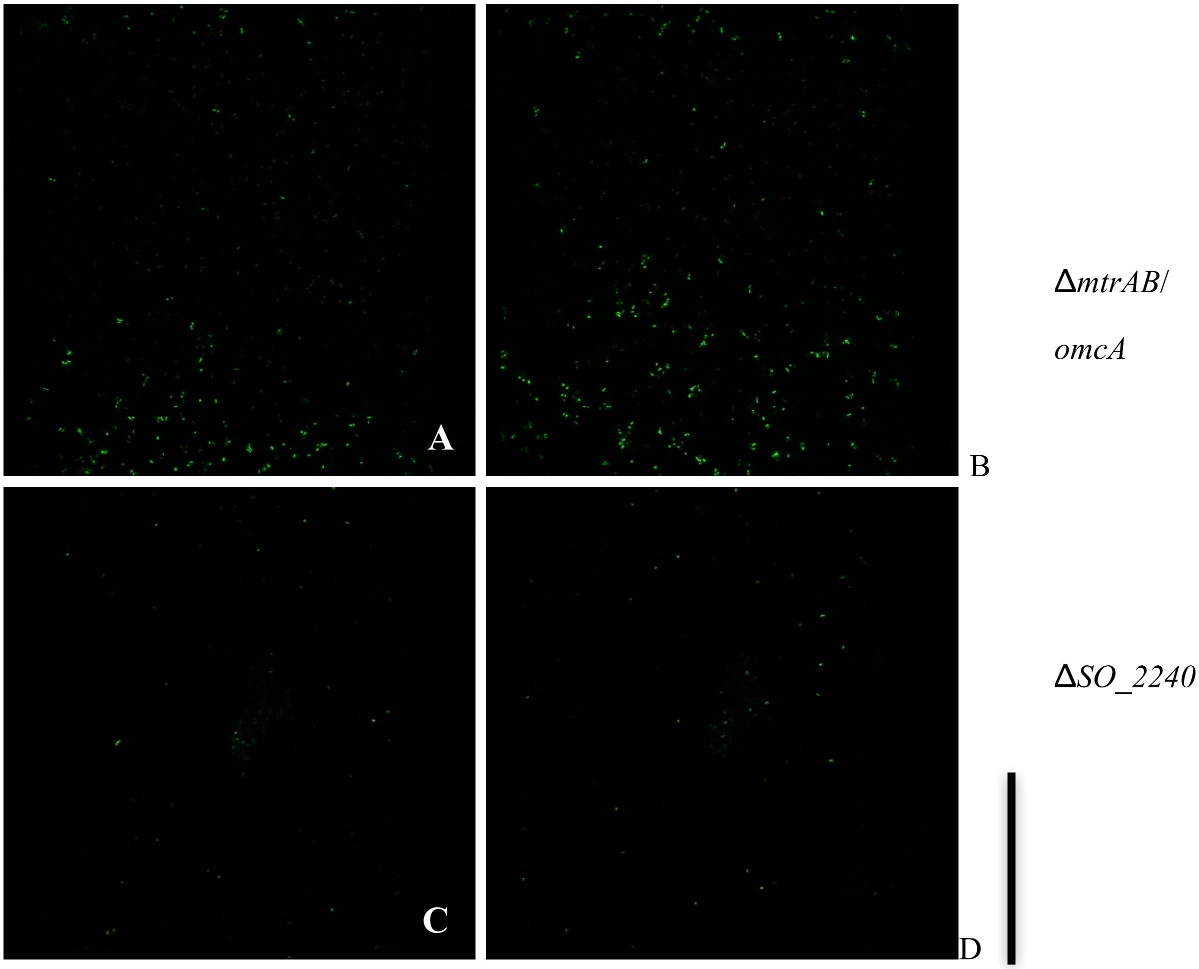

FIG S2. Non-accumulation and attachment of cells on MnO_2 after photo-bleaching. Left column show GFP labeled cells at t = 0 while right column shows experiment at time = 2 h. Experiment with WT MR-1 can be seen in FIG 1. EET deletion mutants Δ*mtrAB*/ *omcA* (A & B) and methyl accepting chemotaxis mutant Δ*SO_2240* (C & D) do not accumulate or attach in significant numbers relative to WT. The black vertical scale bar on bottom right represents 100 μm.
